# Neurofibromatosis Type 1 With Severe Limb Deformity Managed by Above-Knee Amputation With Challenges and Complications: A Case Report

**DOI:** 10.7759/cureus.114643

**Published:** 2026-08-17

**Authors:** Mohammed Tarabishi, Abdullah I Alturki, Majed Alasbali, Abdulmohsin Almehizie, Nada Alsomali

**Affiliations:** 1 Orthopaedic Surgery, King Fahad Medical City, Riyadh, SAU; 2 College of Medicine, King Saud University, Riyadh, SAU

**Keywords:** bleeding complication, limb deformity, major limb amputation, neurofibromatosis type 1 (nf1), vascular anomaly

## Abstract

Neurofibromatosis type 1 (NF1) is a multisystem genetic disorder involving cutaneous, skeletal, neurological, and vascular manifestations. We report a rare NF1 case in an adult male who exhibited typical clinical findings but presented with unexpected intraoperative vascular anomalies. A planned below-knee amputation was converted to an above-knee level due to fragile and dilated vessels encountered intraoperatively, emphasizing the importance of anticipating vascular variations in NF1-related limb deformities.

## Introduction

Neurofibromatosis type 1 (NF1) is a neurocutaneous disease that affects peripheral nerves. NF has two types: NF1 and NF2. NF1 is an autosomal dominant disorder caused by a mutation in the NF1 tumor suppressor gene, positioned on the long arm of chromosome 17 [1]. The gene encodes the protein neurofibromin, which functions as a critical negative regulator of the Ras proto-oncogene (RAS/MAPK) signaling pathway in cell growth [1]. Gene mutation leads to dysregulated cellular proliferation and increased susceptibility to tumor formation. NF1, initially described by Frederich von Recklinghausen in 1882, has a global prevalence of about one in 3000 individuals [2,3]. It demonstrates complete penetrance but exhibits marked variable expressivity.

The diagnosis is based on established clinical criteria, which require the presence of at least two of the following features if the individual does not have a parent diagnosed with NF1 [4,5]. Six or more café-au-lait macules (CALMs), axillary or inguinal freckling (Crowe sign), two or more neurofibromas or one plexiform neurofibroma and optic pathway glioma, two or more Lisch nodules (iris hamartomas), a distinctive osseous lesion (sphenoidal wing dysplasia or tibial pseudoarthrosis, and a heterozygous pathogenic NF1 variant with a variant allele fraction of 50% in apparently normal tissue, such as white blood cells. If a parent is diagnosed with NF1, one or more is enough to merit an NF1 diagnosis. The clinical manifestations of NF1 are multisystemic. Cutaneous features include CALMs and freckling, which often appear in early childhood. Neurofibromas, which are benign peripheral nerve sheath tumors composed of Schwann cells, fibroblasts, mast cells, and perineural cells, are a hallmark. They are categorized as cutaneous, subcutaneous, or plexiform, the latter possessing the potential for malignant transformation into malignant peripheral nerve sheath tumors (MPNSTs) [4-6]. Neurological complications are common and include macrocephaly, learning disabilities, seizures, and an increased risk of central nervous system tumors, such as optic pathway gliomas and glioblastomas. Orthopedic manifestations can include scoliosis, pseudoarthrosis, pectus excavatum, limb deformities, joint instability, and pathological fractures and dislocations [4]. However, indications for limb amputation are rare and typically reserved for severely deformed, non-functional limbs or life-threatening complications [7,8]. Vascular involvement is a significant concern, encompassing congenital heart defects, renal artery stenosis, and other vasculopathies that can lead to secondary hypertension [7,9,10]. Patients may also experience behavioral and psychiatric issues, including anxiety, depression, and social difficulties [11]. 

We report a challenging case of a 35-year-old patient with NF1 who underwent amputation of a severely deformed right lower limb. The procedure was complicated by substantial intraoperative hemorrhage originating from unexpectedly enlarged and friable neurovascular structures, including the femoral artery, vein, and sciatic nerve. This resulted in a sudden decrease in hemoglobin levels, necessitating a massive transfusion. A multidisciplinary surgical team successfully completed the above-knee amputation in an expected manner to achieve hemodynamic control. The postoperative course was notable for the application of a PICO negative-pressure wound therapy system, which facilitated excellent wound healing and an uncomplicated recovery [12]. This case underscores the significant vascular anomalies associated with NF1, illustrates critical intraoperative decision-making, and demonstrates the value of advanced wound management technologies in achieving optimal surgical outcomes.

## Case presentation

**Figure 1:**
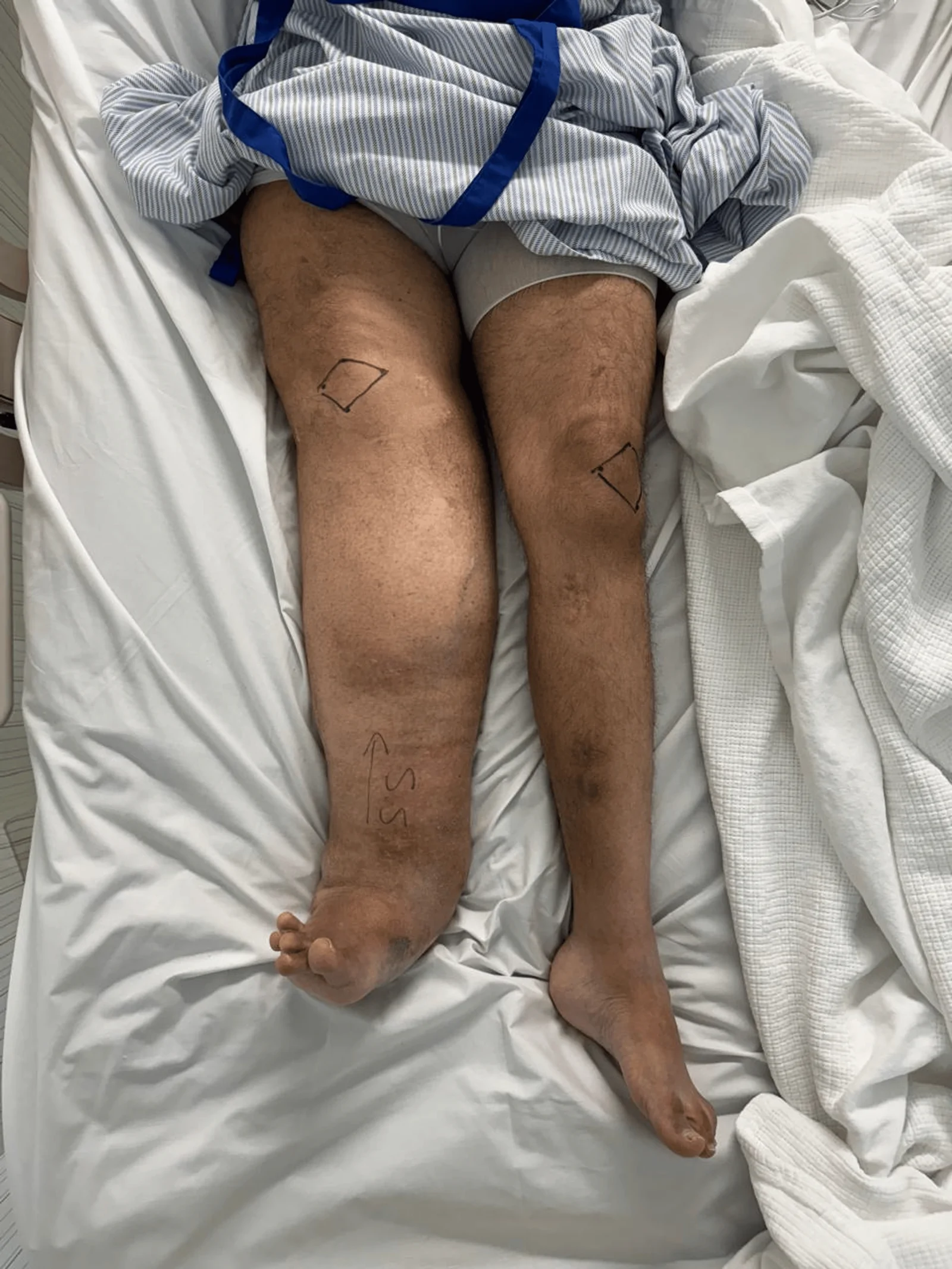
Clinical presentation showing the right lower extremity.

A 35-year-old male with an established history of NF1 presented for an evaluation of progressively worsening motor weakness and instability of the right lower extremity. He had a long-standing neurological hip dislocation, very unstable knees and ankles, and a tibia fracture that had not healed for six years. The limb exhibited massive enlargement, weighing approximately 40 kg, which had resulted in complete loss of ambulatory function for the preceding six years. His medical history was notable for severe osteoporosis, C8-level traumatic tetraplegia, and a prior episode of pulmonary embolism attributed to immobility. Previous surgical interventions included cervical spine fixation and lower extremity soft-tissue releases. Physical examination revealed significant enlargement and shortening of the right lower extremity by approximately 5 to 7 cm compared with the left, accompanied by gross deformity and a large thigh mass consistent with a plexiform neurofibroma (Figure 1). The knee joint demonstrated severe instability, characterized by passive hyperextension to 70-80 degrees and abduction to approximately 20 degrees, with an absence of active flexion. The midfoot exhibited pathological mobility in the medial and lateral planes. The ankle joint also showed limited mobility. Table 1 shows a summary of diagnostic criteria in an individual who does not have a parent diagnosed with NF1.

**Table 1:** Summary of diagnostic criteria in an individual who does not have a parent diagnosed with NF1 Source: [4] CALM: café-au-lait macule, NF1: neurofibromatosis type 1

Characteristics	Criteria
1. Presence of six or more CALM	5 mm in prepubertal, 15 mm in postpubertal
2. Presence of two or more neurofibromas or one plexiform	-
3. Freckling	In the axilla or groin (Crowe sign)
4. Optic nerve glioma	-
5. Two or more Lisch nodules (iris hamartomas)	-
6. Bone lesion	Sphenoid wing dysplasia, typical long bone abnormalities, pseudoarthrosis
7. A heterozygous pathogenic NF1 variant with a variant allele fraction of 50% in apparently normal tissue	-

**Figure 2:**
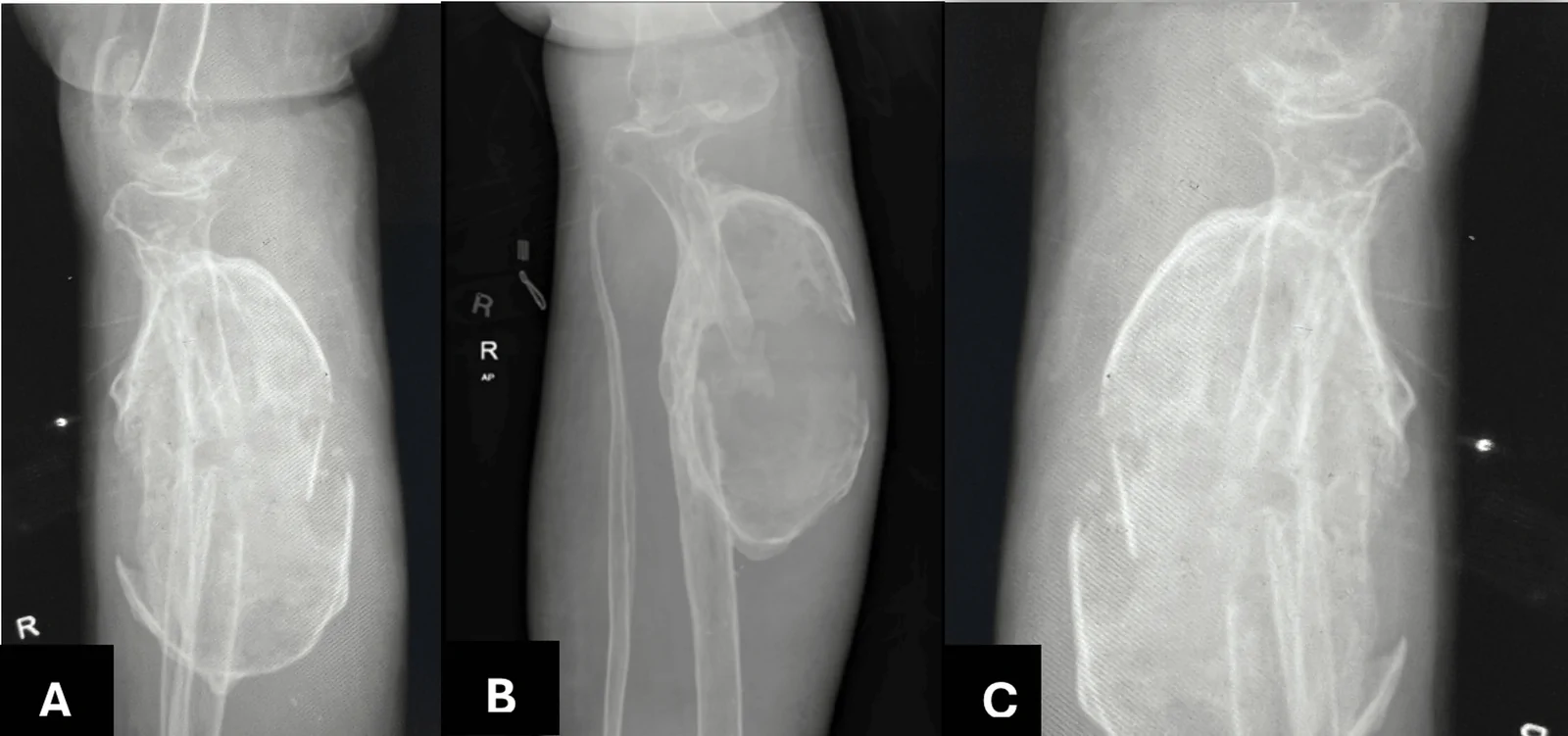
Skeletal abnormalities. Preoperative imaging finding: A, B, and C showing pseudoarthrosis on the tibia.

**Figure 3:**
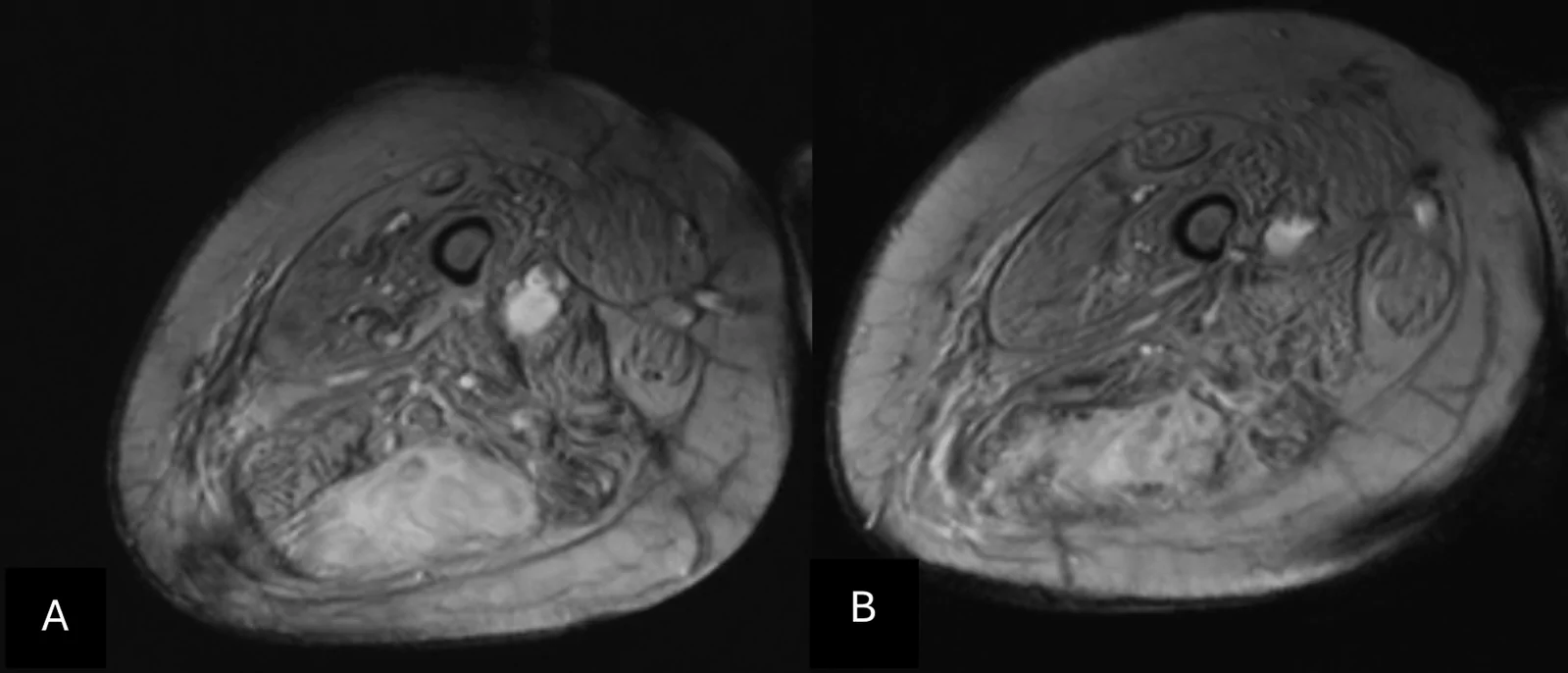
Preoperative MRI of the thigh. A and B show a large plexiform over the posterior thigh compartment, measuring 6.5 x 3.3, and a heterogeneous mass arising from the distal sciatic nerve, measuring approximately 3.5 x 3 cm, in the axial plane, starting from the mid-thigh down to below the knee.

Preoperative X-rays showed leg length discrepancy, hypertrophic ossification, and pseudoarthrosis. Furthermore, there were degenerative changes in the knee joint (Figure 2). Preoperative magnetic resonance imaging (MRI) confirmed a large, infiltrative plexiform neurofibroma involving the thigh; no discrete vascular malformation was identified (Figure 3). Due to the severity of the patient’s deformity and functional impairment, a below-knee amputation was planned under general anesthesia to optimize the functional outcome and quality of life.

**Figure 4:**
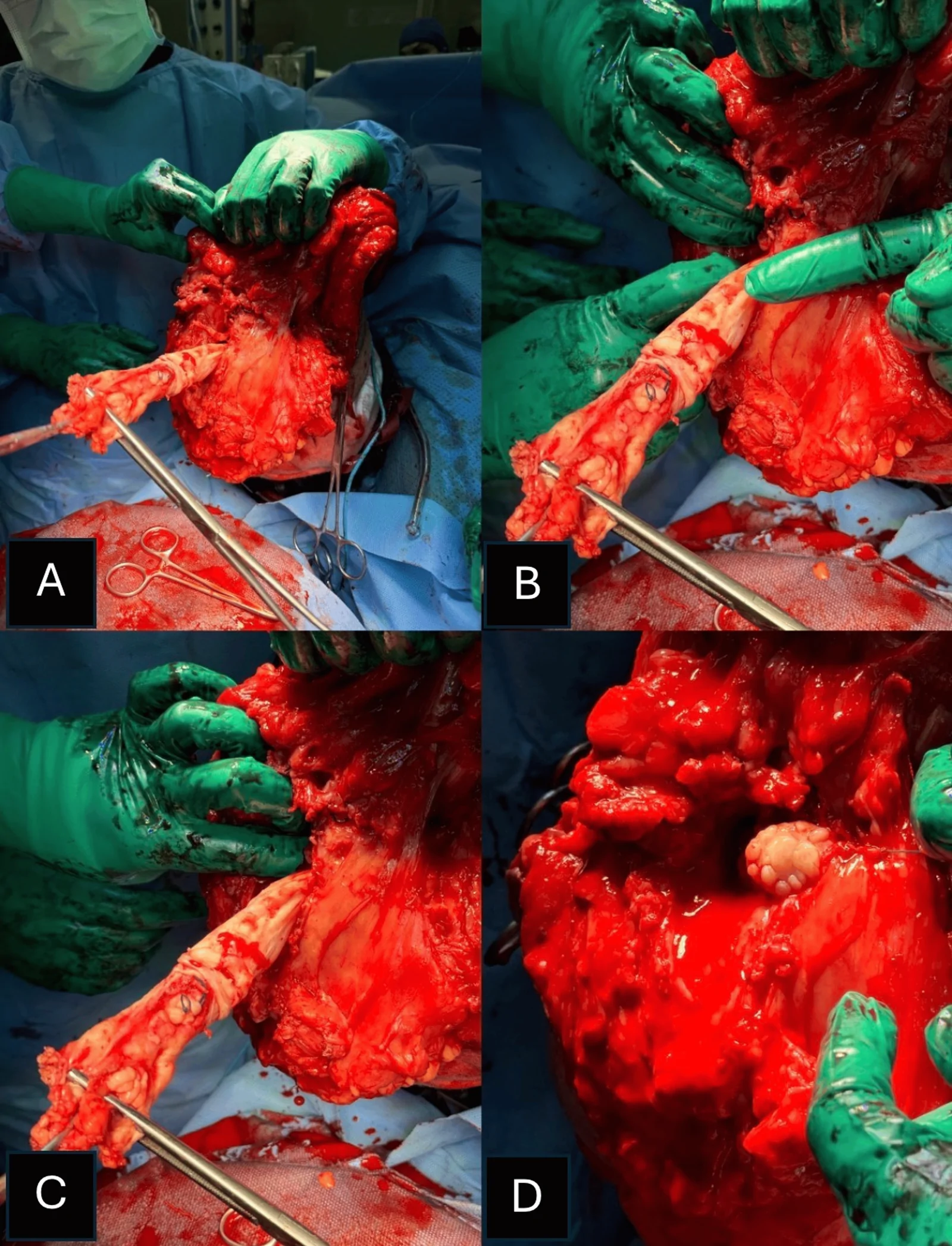
During the amputation, the sciatic nerve was thickened and tortuous. A, B, and C show the large diameter of the sciatic nerve, and D shows how fragile and bloody the cuts were.

Intraoperatively, however, the initial skin incision revealed unexpectedly friable soft tissue with extensive uncontrollable hemorrhage, necessitating a conversion to an above-knee amputation (AKA). Dissection demonstrated markedly enlarged neurovascular structures, including the sciatic nerve, femoral artery, and vein, exceeding 2.5 cm in diameter with attenuated walls, which was the source of massive bleeding (Figure 4). The result precipitated an acute drop in hemoglobin from 11 g/dL to 4 g/dL (normal range: 13.5 to 17.5 g/dL) [13], necessitating the transfusion of eight units of packed red blood cells. A vascular surgeon was consulted intraoperatively, and in a coordinated effort with the orthopedic team, the AKA was completed within 20 minutes to control further blood loss. Hemostasis was achieved via ligation of the femoral vessels, and the wound was closed in layers over a drain system delivering -80 mmHg continuous negative pressure, promoting healing by reducing edema and enhancing perfusion. With daily dressing changes, the wound healed excellently without infection or dehiscence.

**Figure 5:**
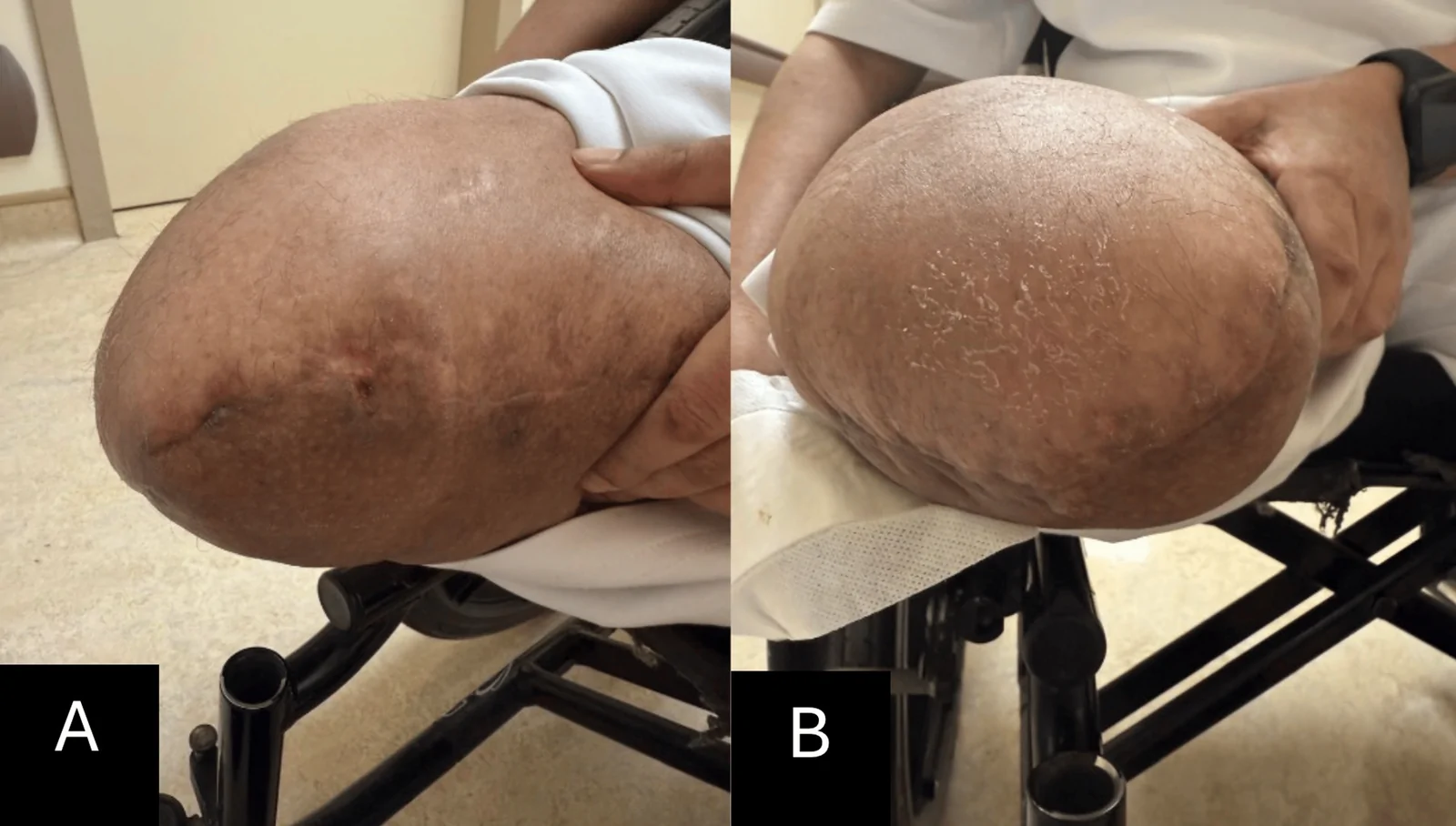
Clinical photographs of the residual limb following amputation, illustrating the postoperative healing process. A and B demonstrate the condition of the amputation stump during the healing phase.

A negative-pressure wound therapy system was applied postoperatively. The patient was transferred from the operating room in stable condition, avoiding ICU admission. The patient was able to transfer back to a wheelchair by the second day after surgery. He was then sent to the prosthetics clinic, where he is being evaluated for a knee-disarticulation prosthesis (Figure 5). The RAND 36-Item Short Form Survey (SF-36) was used to measure health-related quality of life, and the results are shown in Table 2 [14]. This case highlights the crucial importance of surgical adaptability in managing unforeseen anatomical variants. 

**Table 2:** Summary of the RAND 36-Item Short Form Survey (SF-36) scores.

Scale	Score
Physical functioning	70
Role limitation due to physical health	100
Role limitations due to emotional problems	100
Energy/fatigue	95
Emotional well-being	100
Social functioning	100
Pain	100
General health	100

## Discussion

NF1 is a multisystem disorder with different specialties involved. While skeletal deformities and plexiform neurofibromas are widely known, vascular complications are uncommon yet potentially catastrophic [8,15]. The incidence of arterial involvement in NF1 has been reported to range from 0.4% to 7.4%, encompassing arterial stenosis, aneurysms, arteriovenous malformations, and vessel fragility [15,16]. Spontaneous arterial rupture and significant intraoperative hemorrhage have been reported in various vascular territories, highlighting the erratic characteristics of NF1 vasculopathy [17-20]. In the present case, amputation was indicated due to profound limb deformity, instability, and functional impairment. 

During the operation, the discovery of dilated and fragile femoral vessels led to life-threatening hemorrhage, highlighting the importance of anticipating vascular pathology even when preoperative imaging does not reveal significant abnormalities. Previous case reports indicate that NF1 vascular lesions can penetrate vessel walls and increase the risk of rupture, necessitating perioperative planning with vascular surgical support [18-20]. Another notable feature was the use of negative pressure wound therapy (NPWT). Evidence suggests that NPWT reduces edema, enhances perfusion, and lowers infection rates in complex wounds. Meta-analysis of open fractures shows that advanced dressings help wounds heal faster than regular dressings [21]. However, systematic reviews of NPWT in closed incisions have shown more heterogeneous results [22,23]. Despite these limitations, in our patient, NPWT was associated with excellent wound healing, no infection or dehiscence, and early rehabilitation, consistent with reports of its effectiveness in high-risk surgical wounds [24]. Finally, the patient’s quality of life was assessed using the San Francisco (SF-36) survey tool, a validated tool for measuring health-related outcomes [14]. Postoperative scores were remarkably high across most domains, reflecting substantial improvement in psychosocial well-being, independence, and satisfaction with care. This indicates that even intricate and high-risk NF1 cases can have their quality of life restored through amputation, supplemented by multidisciplinary perioperative management and advanced wound care.

## Conclusions

This case report carefully reviewed the patient's history and considered various treatment alternatives. In addition, this case emphasizes the value of surgical preparedness for unexpected anatomical findings and demonstrates how a rapid, multidisciplinary response to intraoperative crises can achieve successful outcomes despite profound surgical challenges. Effective management of NF1 in severely deformed limbs requires a multidisciplinary approach that addresses both the complex vascular and orthopedic challenges. Early recognition and careful surgical planning are essential to optimize functional outcomes and minimize complications in these high-risk cases.
